# Removing Compliance: Interpersonal and Social Factors Affecting Insight Assessments

**DOI:** 10.3389/fpsyt.2020.560039

**Published:** 2020-09-17

**Authors:** Polona Curk, Sándor Gurbai, Fabian Freyenhagen

**Affiliations:** ^1^ Independent Researcher, London, United Kingdom; ^2^ Human Rights Centre, School of Law, University of Essex, Colchester, United Kingdom; ^3^ Essex Autonomy Project, School of Philosophy and Art History, University of Essex, Colchester, United Kingdom; ^4^ Faculty of Special Needs Education, Institute for Disability and Social Participation, ELTE Eötvös Loránd University, Budapest, Hungary; ^5^ School of Philosophy and Art History, University of Essex, Colchester, United Kingdom

**Keywords:** clinical insight, insight assessment, treatment compliance, human rights, therapeutic relationship, supporting insight

## Abstract

This paper probes the format and underlying assumptions of insight conceptualizations and assessment procedures in psychiatry. It does so with reference to the often-neglected perspective of the assessed person. It delineates what the mental steps involved in an insight assessment are for the assessed person, and how they become affected by the context and dynamics of the clinical setting. The paper examines how expectations of compliance in insight assessment tools and procedures extend far beyond treatment adherence, to compliance with diagnostic language and the assessment relationship. Such compliance can be ethically problematic and not in line with human rights standards, notably the Convention on the Rights of Persons with Disabilities. Most importantly, it can be counterproductive in supporting an individual to gain better insight in the sense of self-knowledge. The paper concludes with guidelines for a new approach to insight. This new approach requires taking into account currently neglected components of insight, in particular its relational and social dimensions, through which a person’s insight operates and develops, and through which it could be supported. Concretely, this would mean removing the condition of compliance and reflecting on the influence of the clinician-patient relationship and assessment situation on insight.

## Introduction

Insight is one of the most complex yet influential ideas in psychiatry. Considered clinically relevant since 19^th^ century, it is mainly in the last three decades that a systematic empirical approach has developed. A range of definitions of clinical insight and corresponding insight assessment scales has been proposed (1), and used to examine the thus-defined concept’s correlation to various aspects of illness, behavior, and personality, such as psychopathology, mood, IQ, and adherence to treatment (2, 3). The definitions of insight vary from a more narrowly understood awareness of a particular condition and/or its aspect, to a wider notion encompassing a variety of different types of judgements about what is happening to the individual [(1) pp. 200-201]. In a vibrant and challenging field of research, the notion of insight seems to remain difficult to conceptualize in a consistent way [(1) p. 198]. Nonetheless, as will be shown in the paper, the variety remains circumscribed within the medical model as the main basis of understanding the phenomenon of insight.

The current DSM-V mentions impaired insight over 30 times across various diagnoses, amongst the diagnostic criteria, illnesses’ “descriptors,” “specifiers,” or “associated features.” Given how DSM shapes clinical practice, it is reasonable to conclude that insight is an important clinical variable also in practice. Nonetheless, there is little research on how the concept of insight features in routine clinical practices on wards. The research there is, shows that in clinical settings insight is used with little detail and specificity [(4), (5) p. 2]. Whilst insight tools are rarely relied on [(3) p. 1], there is some evidence that psychiatrists’ accounts of insight are still informed by the dominant^1^ psychiatric literature. In one of the rare studies of the practitioners’ views (6), the interviewed psychiatrists referred to insight as a “sense of illness,” understood to be related to a kind of “objective,” “rational,” or “critical” knowledge informed by psychiatric discourse, which they, in turn, expected the patients to have or be willing to acquire. Their accounts invariably constructed insight as proved by treatment compliance^2^ [(6) pp. 1462-1464].

Despite not being a legal term, the concept has also played a central role in mental health and mental capacity legal settings, where psychiatrists’ views have often had a decisive influence on outcomes. The term “insight” is mentioned in judicial decisions in England, Australia, New Zealand, and Sweden, amongst others (7–9). As Kate Diesfeld has highlighted in a study of mental health tribunals, liberty- and human rights-affecting decisions often come down to clinicians’ perceptions on insight (7). Diesfeld cites studies, which have shown that, (a) in some cases perceived lack of insight was the only observed symptom on which the decision at the tribunal hinged, and (b) non-compliance with treatment was the most frequent justification for refusing discharge. The concept of insight has also been relevant in mental capacity settings, such as in England and Wales’ Court of Protection cases (10, 11). For example, as shown by Paula Case, expert witnesses—psychiatrists in more than 50% of cases—frequently import clinical terminology such as of “lack of insight” and “compliance” into their statements about assessing not only the diagnostic but also the functional part of the decision-making capacity.^^3^^

The important role the concept of insight has played in legal settings has given rise to a number of ethical and human rights-based concerns. For example, in the above-mentioned study, Case is concerned that importing clinical terminology into legal settings in a way that is determinative of legal outcomes, may undermine the autonomy-promoting provisions of the Mental Capacity Act 2005 [(10) pp. 361-2]. Case may have a point: there is evidence that psychiatrists understand insight as strongly (2) or even causally (12) connected to mental capacity, even when they have established only a correlation between them. The already mentioned study of mental health tribunals provides another example: it was found that there was rarely any evidence provided for how impaired insight had been determined by the clinicians [(7) pp. 362-365].

Some have argued that loose and unspecified use or short-handing insight for mental capacity is not an intrinsic fault of the insight concept [(3) p. 2]. But this paper argues that, quite beside the disagreements about its (unclear) use in legal and medico-legal settings, certain aspects of current insight conceptualizations and assessment procedures in both research and clinical practice, may themselves already be problematic, and looks at why this may be relevant for the care someone is provided as well as in the human rights context.

The pervasiveness of the concept indicates that the term insight captures something important in respect to understanding someone who seems to have a radically different view of some aspect of their condition or experience. It can, thus, be a useful construct when used wisely (3), for example to find the best way for their care [(5) p. 5] But it needs a careful approach and an inclusive understanding of the phenomena.

The paper makes two main arguments. The first argument is that underlying assumptions of compliance extend far beyond treatment adherence, to compliance with medical discourse, and finally, with the assessment procedure, context, and relationship. The paper thereby introduces a wider notion of compliance than is operative in the existing literature. Crucial for this are two moves the paper makes:

- It highlights how insight, instead of indicating self-knowledge/self-reflexivity, becomes equated with whether a person complies with the medical understanding of their experience. This can illegitimately reduce important ethical and legal decisions to medical ones. Psychiatric research literature and actors in legal settings invoke both understandings, and sometimes run them together in a way that conceals the compliance demands built into psychiatric conceptualizations and practices, removing them from the critical scrutiny in light of human rights and wider ethical standards that they should get. Social and relational dimensions of a person’s insight are not systematically included, or not considered at all.
- The paper develops an original analysis of the mental steps required for the assessed persons in an insight assessment and how they may be affected by the required compliance with the context and dynamics of the assessment procedure and relationship.

The second argument is that as insight understood in the broad sense of self-knowledge/self-reflexivity can be fundamentally affected by the relational dynamic of the assessment, supporting it is incompatible with the context of compliance and power asymmetry in the insight assessment procedure. In other words, the situation of compliance and asymmetrical power relations is *especially* problematic in the context of exploring insight into one’s mental states and experiences because it can adversely affect it. The paper argues for a shift of perspective not only in regard to how insight should be considered—so that the influence of the assessment interactions is appropriately included in the assessment—but what assessment of insight can be for—as a basis for support rather than risk assessment or prevention.

We build on these two arguments to develop suggestions for how insight could be approached differently. Drawing on a case vignette, we first illustrate one way in which insight can be relationally supported, before developing guidelines for such a support-orientated understanding of insight. We also indicate that this understanding would better meet human rights requirements, in particular moving to the supported decision-making paradigm, than existing conceptualizations and practice.

The paper is structured as follows: the first section briefly summarizes the existing debates around inclusion of treatment compliance in insight conceptualizations and scales, and discusses whether relying on good practice is sufficient to avoid the problems this inclusion can give rise to. The second section shows that insight conceptualizations and scales also require compliance with medical discourse. It exposes an oscillation between the broad and narrow notion of insight which has problematic implications in terms of ethics and human rights. In the third section, we analyze the steps taking place in the insight assessment procedure, to demonstrate that insight is assessed through an interactive engagement, requiring mental steps that are dependent on the nature and quality of the assessment situation, context, and foremost, relationships. Here, too, the standard approach builds in compliance, as it allows only a specific configuration of this interaction. The next section presents insight as an interactive, dynamically negotiated construct. It provides an example of what supporting insight as self-knowledge involves. The paper concludes with guidelines.

## Treatment Compliance

Despite the variety of proposed insight conceptualizations and scales, there are some common features. Insight is understood as multidimensional, and its main proposed components can be summarized as, broadly speaking, (a) an awareness of mental illness and symptoms, (b) awareness of the need for treatment. Whilst some scales additionally include (c) understanding the implications and social impact of the illness, and ability to plan, and (d) distinguish awareness of symptoms from their attribution to illness, Ken Kress (13) argues that there are in fact predominantly only two main dimensions of insight in mental health: (1) recognition of mental illness and (2) recognition of the need for treatment. However, the inclusion of (b)—and specifically treatment compliance as evidence for it—as a component of insight, has been controversial, both from a conceptual and epistemological perspectives.

One of the first instruments devised to systematically measure lack of insight was Item G12 on The Positive and Negative Syndrome Scale for symptoms of schizophrenia (PANSS), published in 1987 (14). Item G12 describes seven levels for lack of insight. The criteria for the penultimate level 6, which describes lack of insight as “severe,” is that the patient denies ever having had a psychiatric disorder or symptoms or the need for treatment *but* is nonetheless compliant with treatment. It is lack of treatment compliance that brings one to the top level 7: “extreme” lack of insight (15). In other words, the scale proposes that if you do not believe you have a disorder or need medication, it is more insightful to comply with treatment anyway, than to not comply. Conceptually speaking, this seems strange. Nonetheless, most other main scales, such as ITAQ, SAI-E, and SUMD, also give points for treatment compliance and negative answers will be scored as lower insight (16–18). On these scales, agreeing to treatment is *always* more insightful than not to.

This seems to miss out on capturing the option that, as it seems intuitively clear, an individual’s views on treatment can be both positive and negative, and perhaps *both*, unrelated to insight. For example, rejecting pharmacological treatments can have other reasons, such as side effects, wanting to weather things out, trying alternative treatments, exploring one’s madness, or even liking certain aspects of it. Rejecting psychotherapy treatments can be because one does not want to examine something traumatic or speak about emotions. These can all be at the core of completely insightful perspectives. One does not have to want to have treatment to be insightful. Whether having treatment is more insightful can be a matter of a debate just as whether one should stop smoking, eat less, exercise more, or follow other health advice.

Perhaps it goes without saying that in practice, or at least in good practice, the insight assessor will recognize such reasons as insightful although the instruments themselves do not. However, underneath hides a neglected presupposition: it is inevitable that it will be the clinician who makes a distinction between an (insightful) reason for refusing treatment—say, side effects, which is a recognizable term and phenomena for the clinician—and a (presumably not insightful) one, say, that medication is poison (which a trained psychiatrist might have more difficulties to accept as reasonable). For example, a clinician might judge that it is simply not valid to not believe in medication, or to not want to take it for no particular reason at all (despite the fact that some people do not want to stop smoking without offering any reason for it, and are permitted to do so). Even in good clinical practice, it is left to the discretion of the psychiatrist to decide which are valid or invalid reasons. Depending on the legal safeguards, the discretion of the clinician might be qualified by the requirement that two clinicians agree, and by the right to appeal, such as in England, to Mental Health Tribunals. But the basic point remains: the validity is adjudicated by a clinician or group of clinicians, at least until challenged by way of legal review procedures.

Thus, despite the good practices that may go beyond what the instrument expects, including treatment adherence into insight conceptualizations remains problematic: it violates one’s right to decide not only about one’s own treatment, but also about one’s own values. In the case where the clinician’s view on treatment differs from the patient’s, it may expose the latter to suffering detriments in human rights, such as (unjustified) deprivation of liberty, denial of the right to respect for physical and mental integrity, or loss of the right to privacy. In addition, some instances of bad practices and non-accidental risks for such bad practice are already sufficient for worries regarding human rights requirements.

Besides conceptually, treatment compliance has been a contentious component of insight also from epistemological perspective. Although it has been studied extensively, disagreement persists: some believe a consensus has been reached that insight is a predictor of treatment adherence [(3) p. 1], while others claim the studies produced inconclusive results (1). The relationship between one’s understanding of one’s experiences and one’s decisions about treatment is complex. The reasons for the complexity stem partly from the mind’s different layers of awareness and tendency to allow for contradictory states and beliefs. As David cautions: “People will accept and even welcome treatment while still maintaining that they do not have an illness and vice versa.” (19) Secondly, further complexity is added by how profoundly and *continually* we are influenced by others in our sense-making and our decisions. As McEvoy et al. recognize, “[it] may be that inpatient compliance more immediately reflects the socialization of patients to expected behaviour than any clear recognition of the need for treatment” [(16) p. 46]. Greenfeld et al. imply that simply what kind of questions have been asked matters in regard to results in research on insight [(20) pp. 250-251].

This already points to the importance of the procedure, context, and relational issues in insight assessments, which we will turn to further below. But before that, let us look at whether the issue of compliance is resolved if treatment compliance is removed from conceptualizations of insight.

## Compliance With the Medical Model

In response to qualms about treatment compliance as an insight component, some have offered alternative approaches. One proposed solution has been to focus on treatment benefits for the patient (removing the focus on the acceptance of a particular diagnosis) (21). However, this brings its own problems, for example, that the person will not get the right care and support in the current system, or getting into a kind of “uninformed consent” dilemma [(22) p. 85]. On the other side, Markova et al.—concluding that there are no theoretical grounds for considering treatment compliance intrinsic to the insight concept [(23) p. 86]—removed items referring to treatment from their 1992 scale (see **Table 1** for the features of the main insight scales, including the Markova et al.’s one).

**Table 1:** Features of the main insight scales and issues of concern.

Insight instrument	Features of the instrument	Issues of concern
Item G12 on the Positive and negative syndrome scale for schizophrenia (PANSS) (14)	1) Definition/components: Lack of insight is defined as impaired understanding of one’s own psychiatric condition and life situation failure to recognize psychiatric illness or symptoms, denial of need for hospitalization and treatment poor anticipation of consequences and unrealistic planning Seven levels of severity of lack of insight. Denial of psychiatric illness or symptoms and denial of need for hospitalization and treatment are considered as severe lack of insight; refusal of medication is considered an extreme lack of insight. 2) Attitude in relation to both past and present experiences are considered.	Being rated as having insight requires acceptance of psychiatric discourse, medical treatment, and cooperation with therapists. The paper accompanying the scale judges disagreement with the clinician and hostility towards her/him, sarcasm, uncooperativeness, or disrespect, as part of pathology. The instrument does not take into account that some of the items measured (for example, tension, anxiety, etc.) may be partly a result of the interview situation itself rather than pathology. It does not account for socio-cultural background, or require self-reflexivity on the part of the clinicians such as about social distance to the patient and the quality of the relationship. Treatment refers to hospitalization and psychiatric treatment only. Explaining the need for treatment in relation to other (non-psychiatric) problems, such as anxiety, tension, or sleep difficulty, is considered “rationalization.” Only full acceptance of the psychiatric model is considered insightful. Denying illness but taking medication anyway is rated as more insightful than denying illness and refusing medication.
Insight and Treatment Attitudes Questionnaire (ITAQ) (16)	1) The insight questionnaire tries to establish: Recognition of mental problems Recognition of need for hospitalization Recognition of the need for treatment with medication Recognition of vulnerability to recurrence of problems Attitude to medication and intent to take it after discharge 2) The scale asks about mental problems, need for hospitalization, and need for medication in relation to past and present experience, and in relation to the future. 3) The scale asks the person for an explanation in relation to their answers.	Being rated as having insight requires that the patient judges their experience as pathological in a manner congruent with the clinicians and that they believe they need treatment. Treatment refers to hospitalization and pharmacotherapy only. The instrument does not account for socio-cultural background, or require self-reflexivity on the part of the clinicians such as about social distance to the patient and the quality of the relationship.
Schedule for the Assessment of Insight (SAI) (17)	1) Insight has three components: Recognition of mental illness Compliance with treatment (passive and active) Ability to attribute symptoms to illness The three dimensions are overlapping. Insight can be partial. 2) If an explanation of illness is given that is considered appropriate to the socio-cultural and educational background, points for insight can be given. 3) Treatment refers also to other physical and psychological therapies.	Being rated as having insight requires acceptance of having a (psychiatric) illness, acceptance of treatment, and attribution of experiences to illness. The scale advises that an appropriate explanation for illness given the person’s socio-cultural and educational background can be considered fully insightful, including, for example, stress, family history, etc. However, in the next section, if a particular experience is understood as a reaction to outside events, such as stress, tiredness, etc., this is considered only partially insightful. Full insight is only established if a medical view is adopted by the person in full. It does not require self-reflexivity on the part of the clinicians such as about social distance to the patient and the quality of the relationship.
Scale to Assess Unawareness of Mental Disorder (SUMD) (18)	1) The scale has a 17-item symptoms list to be completed prior to the scale. The scale consists of three summary items: Awareness of mental disorder/psychiatric problem Awareness of the achieved effects of medication Awareness of the social consequences of the mental disorder After that, awareness and attribution of the relevant ones from the 17 symptoms are assessed. Insight can be partial. Insight is “modality-specific”: it can vary across various manifestations of the illness and can be present in relation to a particular symptom but not another. 2) Awareness of each item and symptom is assessed in relation to the current experience and to past episodes.	Being rated as having insight requires acceptance of psychiatric discourse. Treatment refers to medication only. If the person believes the medication has not lessened their symptoms, they are considered unaware. No other belief or explanation in regard to treatment is considered. Although in the accompanying paper the authors recognize that what constitutes a sign or symptom of a mental disorder may vary widely from one culture or subculture to another, this is not reflected in the scale and is (presumably) left to the clinician’s opinion. The scale does not require self-reflexivity on the part of the clinicians such as about social distance to the patient and the quality of the relationship.
Insight in Psychosis Questionnaire/Insight Scale (IS) (23)	1) Insight is described in relation to an experience of change: Experience of change Domain of perceived change (within the self, the environment, or both) Focus of change (thoughts, feelings, etc.) Attributing the perceived changes to illness Practically oriented—the scale is based on a wider concept of insight as self-knowledge, knowledge of the illness and how it might affect his/her ability to function within the environment. It focuses on the subjective experience of the patient and his/her relationship to the environment, rather than diagnosis or labeling. 2) Items relating to views about hospitalization or medication were deleted in the new version.	Although the references to the need for treatment and hospitalizations were removed, the scale still scores points for insight on the basis of admitting that one is “ill,” there is something “wrong,” and one feels different from “normal.” Attributing experiences to “feeling tired” or similar are not scored as insightful. The scale does not account for socio-cultural background, or require self-reflexivity on the part of the clinicians such as about social distance to the patient and the quality of the relationship.

When we remove the treatment compliance component from insight assessment, the focus turns to the awareness of illness. Markova et al.’s new scale focuses on individual’s experiences. Nonetheless, requiring explicit agreement with the idea of illness is still there. Scoring points for insight requires agreement with statements such as “I am ill” and disagreement with statements such as “There is nothing wrong with me” [(23) p. 87]. Other scales frame the awareness in even more explicitly medical terms:

“1. At the time of admission to this hospital, did you have mental (nerve, worry) problems that were different from most other people’s? Explain.” [(16) p. 47]

“2a. Ask patient: ‘Do you think you have an illness?’” proceeding to:

“2b. Ask patient: ‘Do you think you have a mental/psychiatric illness?’” proceeding to:

“2c. Ask patient: ‘How do you explain your illness?’” [(17) p. 806]

“1. In the most general terms, does the subject believe that he/she has a mental disorder, psychiatric problem, emotional difficulty, etc.?” [(18) p. 879]

The scales differ in the terminology, referring to mental illness, disorder, mental worry, psychiatric condition, and other. However, all above-mentioned scales give points for insight when the individual in question endorses having an illness and/or agrees with the diagnosis. The issue of expecting compliance in relation to insight turns out to be wider than one might have thought.

It is clear from the above that the most frequently used insight scales need a diagnosis for the assessment to make sense. First, a presence of symptoms is established and a diagnosis given, then the individual’s appreciation of their experiences is examined. The instruments do not aim to assess whether one is mentally ill: they expect that to be a given.

As a result, the scales are insensitive to distinguishing between healthy control groups and people with mental illness. As David et al. [(24) p. 1384] put it: “…asking a self-aware healthy person to perform the metacognitive task of saying whether they suffer from a mental illness or symptoms thereof should lead to an emphatic ‘no’, while the patient with SCZ who lacks all insight into their condition will give the same response, with the same certainty.” Beck Cognitive Insight Scale (BCIS) (25) attempted to remedy this by proposing “cognitive insight,” which refers to one’s ability to evaluate and correct one’s beliefs through the sub-scales of self-reflectiveness and self-certainty. However, as David et al. (24) note, BCIS still refers to unusual experiences, which not all might have (or consider “unusual”). In sum, if there is not a *prior* diagnosis, or judgment of there something being wrong, pathological, or at least “unusual,” insight instruments do not make sense.

This might still not seem problematic, unless one wants to problematize psychiatric diagnoses in the first place, which is not what is attempted here. However, even if the relevant conclusions about clinical insight are “as close to being ‘facts’ as anything else in the clinical psychiatry” (3), the issue that insight scales do not make sense without diagnosis exposes problems with their underlying conceptualization of insight.

As a quick detour to help elucidate this, let us look at the use of the word “insight” in Court of Protection cases. It shows not only that the concept is used both in clinical and generic (non-clinical) senses of the word, but also it is not always possible to determine whether clinicians and judges are referring to clinical insight in particular. For example, besides P’s insight into their impairment or needs, which might be considered as referring directly to “clinical insight,” the term is also used to refer to:

- P’s insight into others’ needs;
- P’s insight into effects of P’s actions on others;
- P’s insight into her personal relationships;
- P’s insight into P’s own personality traits and talents;

All these are not usually part of insight assessments. In addition, the term insight has even been used in court in reference to the carer’s understanding of P’s needs (26–30).

This signals that there are two notions of insight in play in psychiatric research and practice as well as legal settings. As mentioned at the beginning, the conceptualizations of insight range from a narrower awareness of particular aspect of illness to a wider idea of self-knowledge. For the sake of clarity, **Table 2** provides orientating definitions of the two notions of insight.

**Table 2:** Two notions of insight.

*Narrow notion of insight*	Insight is a matter of awareness of illness (or impairment), where what counts as illness (and impairment) is, ultimately, understood in terms of the medical model.
*Broad notion of insight*	Insight is a matter of self-knowledge, where what counts as self-knowledge is, ultimately, understood in terms of the level of self-reflexivity a person has about their experiences and conditions.

There is a crucial move at the basis of insight conceptualizations in psychiatry, through which insight (which in the case of a carer above, for example, would simply describe how she understands her charge’s needs) becomes equated exclusively with whether a person agrees with the medical understanding of their experience.

Let us consider a few examples to help explain why this is problematic. This is a recent statement in a paper that defends insight conceptualizations in psychiatry:

“Insight is another word for self-knowledge and it may even have a dedicated brain system for its implementation. It is more about a conversation we have with ourselves about what we truly believe than the opinion of others. Acknowledging that one is ill or impaired or in need of help is never easy but, we contend, it is necessary for living an authentic life.” [(3) p. 2].

First, insight is equated with self-knowledge; then, there is an implicit shift from the broad notion of insight (self-knowledge) in the first sentence to the narrow notion (awareness of illness or impairment); and, finally, this narrow notion is linked with an ethical value (authenticity) that is not directly linked to medicine.

One might think that a shift from the broad notion of insight to the narrow one is innocent and natural, given the context. When self-knowledge might include a number of dimensions, one might think the dimension that is relevant in the context of psychiatry—and in the related legal settings—is about self-knowledge about one’s mental illness or impairment^4^. Many have expressed concerns that the scales then essentially measure a discrepancy between the assessed individual and the clinician (5, 31) or in relation to a “gold standard” (24), a concern discussed in relation to insight since Jasper’s and Lewis’s definitions of “correct” attitudes and judgments [(17) p. 799] [(32) p. 333]. It may also be methodologically suspect: the measuring tool expects an alignment of the result with the person making the measurement. As Guidry-Grimes and others have pointed out, it is also problematic because of the power imbalance between the clinician and patient (5), inconsistent or inconclusive results in insight research for example in relation to treatment adherence (1, 5), cognition (33), and certain psychiatric conditions (5); and simply because psychiatry is not infallible (5). Despite these concerns, it might be considered completely legitimate for people highly trained in a specialist body of knowledge to assess whether a lay person complies sufficiently with the gold standard produced by this body of knowledge.

Even if so, the problem we highlight remains. Independently of the standard of correctness for this discipline, the nature of mental illness, or psychiatry as a medical science, there are compliance assumptions underlying the psychiatric insight concept coming from the conflation of the two notions of insight. The main and explicit operative notion in psychiatric research and practice is the narrow one (as shown above, the insight instruments need a diagnosis for the assessment to make sense). However, this notion is used alongside the broad notion of insight and sometimes conflated with it. It is the broad notion that not only covers up the compliance requirement in the narrow notion but gives the concept its weight in legal and medico-legal settings.

This is most clearly visible at the intersection of psychiatry and law. In the context of mental health tribunals and courts deciding about mental capacity, it is reasonable to suppose that it would make a significant difference if instead of psychiatrists’ stating that the persons in question lack insight, such statements were disambiguated to say solely that the persons are not complying with the treatment recommendations or discourse or assessment situation derived from the medical model of psychiatry. Such a disambiguated statement might still be relevant and useful for tribunals and courts, but it is reasonable to expect that it would not be as decisive in determining the legal fate of the individuals in question than stating that they lacked insight (something that, as noted in the *Introduction*, is often the sole decisive factor). The latter statement might be easily misunderstood to be a claim about their general capacity for self-knowledge and self-reflexivity, which includes other considerations (such as, for example, authenticity of life or what is necessary for it), outside of medical or psychiatric matters. Instead of drawing attention to the reasons why a person might not comply with medical treatment, discourse, and assessment relation, a statement about insight that is not disambiguated between the narrow and broad notion of insight might settle important legal and ethical matters by way of an equivocation, such that the expert witness is warranted only to speak of the narrow notion of insight, but is understood to invoke the broad one.

Therefore, it these instruments are to be considered “*insight* assessment scales”—then they should be valid and working for anyone whose *insight* into their mental states and experiences can be assessed, *regardless of what these mental states and experiences are*—so, also for people without a diagnosis or illness. Insight would simply mean a reflection on one’s own mental processes (or indeed, as in the case of the carer, above, another’s mental processes): that is, referring to (self-) knowledge. Assessing a person’s, or a patient population’s, reflexivity and understanding of their experiences might then be primarily an exploratory rather than evaluative endeavor—”*how* do they understand them?”

If they are, on the contrary, instruments to measure how much or how little a patient *accepts* they have an illness in a medical sense for which a medical treatment is the only or most appropriate, then they are, rather, “*agreement with medical view* assessment scales”. In this second case, a) these assessment scales do indeed need a diagnosis prior to the assessment (otherwise there is nothing to measure the agreement with), but it needs to be taken into account that b) you cannot score high on the “*agreement* with medical view scale” if you *disagree.*

If the scales were actually referring to self-reflexivity/self-knowledge (insight in the broad sense), they would work for anyone whose insight can be measured. In contrast, the underlying conceptualization of insight of existing insight instruments is based on conflation that uses a concept that implies one understanding (self-knowledge) but in fact refers to a narrower notion (measuring whether the person accepts the medical understanding of their experience). This is a conceptual problem with the scales that is mostly independent of what we think about the nature of mental illness, or the validity of the diagnosis.

This problem touches on ethics and human rights, once we take into account how the medical model often diverges from other approaches to mental difficulties, while ethics and human rights require considerable respect for the other approaches. As some of the recent literature highlights, both insight as a phenomenon and its assessment are strongly influenced by socio-cultural factors. A study of triads (patient, family member, clinician) by Tranulis et al. (31) has shown that when comparing the patient’s insight into their condition with a family member’s insight into the patient’s illness, they were very closely correlated although the latter were not suffering from illness. Both patients and their entourage were creating explanations for the experiences using their shared background, life experiences, and views on stigma: In Western participants, meanings were constructed around stress, drugs, overwork, whilst immigrant participants spoke about spiritual forces and religion. The authors conclude that “[e]xplanations congruent with the person’s culture should be accepted as evidence of good insight” [(31) p. 238].

There are some important acknowledgments in the insight scales literature of the influence of socio-cultural factors on insight. The authors of SUMD speak about the consistency of the patient’s views with his/her culture [(18) p. 874] and the SAI scale refers to the plausibility of the patient’s beliefs in relation to their socio-cultural milieu (17). However, first, what is considered “plausible” is left to the clinician, and second, it is unclear what happens when this milieu includes something that the clinician does not believe in. There are no guidelines in the procedure about how such beliefs are to be taken into account. Perhaps the expectation is to educate the patient towards medical explanations, as the study of psychiatrists’ account of insight mentioned above suggests (6).

Some have proposed that what can be considered insightful could be more inclusive, for example, a simple recognition that one has problems which affect one’s interpersonal relations and will likely continue without treatment adherence [(22) p. 88], or an acknowledgment of some kind of change in the self that affects social functioning and feeling the need for restitution, but “irrespective of the attribution and the pathways of care that the person seeks,” [(34) p. 108]. However, despite these views, systematically and consistently considering insight as irrespective of attribution of experiences to mental illness, inclusive of other explanatory models, and recognizing as insightful also a person seeking non-medical treatments, seems the opposite of what the dominant conceptualizations of insight and scales allow.

Thus, whilst mental health professionals and researchers may agree about the importance of the socio-cultural factors and declare to be open to other pathways of care, it is unclear that this happens systematically in practice. As with recognition of need for treatment (above), the validity of views diverging from the medical model is adjudicated by clinicians. Ultimately, we are faced with another instance of compliance.

This is important in relation to biases that some report in studies on insight (as measured by insight scales) in relation to social and demographical factors [(1) pp. 78-83]. Moreover Tranulis et al.’s study found evidence that the social distance between the background of clinicians and patients led to clinicians more readily treating certain patients pharmacologically because they felt uncomfortable engaging with the beliefs and ideas of those patients [(31) pp. 228, 234-237]. This suggests that, despite some important mentions of socio-cultural diversity in the literature, the demand for compliance with the medical model built into the scales remains problematic from legal and ethical perspectives.

In the ethical and human rights context in which medical (including psychiatric) care operates, it is widely recognized (at least in liberal jurisdictions) that the best interests of a person are not exhausted by what is medically in their best interests. Thus, even if medically trained professionals are typically best suited to judge what is in a person’s *medical* best interests, this does not settle the question of what is in their overall best interests. Settling this question is not something that medically trained professionals have specific expertise in and, more importantly, their training and expertise gives them no special authority in relation to this wider issue. Moreover, the value of autonomy also comes into the equation: the person should be allowed to determine what happens, even when they are not choosing the course of action in their best interests (both medical and overall best interest), as long as they are mentally competent (which is a necessary and perhaps sufficient condition for autonomy).

More recent developments—notably the United Nations Convention on the Rights of Persons with Disabilities (CRPD)—have affirmed that the rights in question should also apply to persons with disabilities and that any restrictions in these rights should be disability-neutral (i.e., made on a basis that applies to every person, irrespective of disabilities). Otherwise, these restrictions would be discriminatory.

Thus, if lack of insight is accepted as a ground on the basis of which it would be ethically permissible and compatible with human rights requirements to restrict the rights of the person in question, it would have to be the broad notion. Moreover, any assessment of it would have to be extended to everyone, not restricted to groups of protected characteristics, such as people with mental illness. Psychiatric notions of insight, since they depend on a diagnosis and are not valid for healthy controls, would therefore be excluded, otherwise there would be concerns about direct discrimination. This has nothing to do with epistemic worries about psychiatry. Instead, it is due to the fact that compliance with the medical model of psychiatry is not something that can be legitimately required of people when it comes to any restrictions of their individual rights for their own good.

Modern liberal ethics and human rights require that such restrictions and indeed any safeguarding deployed to ensure that autonomy is in place be based on reasons that the person can endorse, thereby respecting their rights, will and preferences [(35) p. §12(4)] Psychiatry’s views on how a person understands herself do not by themselves settle that the person takes them as determinative of, or necessary for, what counts as self-knowledge and self-reflexivity. Whilst the judgment whether a person is compliant with medical discourse is something that would be permissible to report to courts and tribunals, the question of whether or not this means that the person lacks insight in the broad sense, would have to be left open by psychiatric expert witnesses.

## Compliance With the Assessment Relationship

One might ask how one could assess someone’s insight without judging it against the (medical) mode of the clinician, or some accepted “correct” or “gold” standard. We hope to provide some answers in the rest of this paper.

One answer might be, as we saw above, to include the ratings by a caregiver or family member. David et al. (24) caution that this poses a similar problem of a gold standard, but note that the methodology is consistent in showing lower estimate of deficit by patients in comparison to caregivers. Whilst certainly a valuable methodology, even when a person’s view of their experiences or needing help differs from that of their family or peers, this is not conclusive about someone’s lack of insight. The methodology of simply looking for discrepancy is not sensitive to potential mediating variables, for example, emotional motivations of each of these groups behind their estimates, such as tiredness of the care givers, their risk aversion in comparison to the patients’ one, their sense of responsibility, etc.

This highlights another presupposition that the existing insight conceptualizations and scales share: that insight is something intrapersonal. Whilst it can have among its generative conditions socio-cultural factors, none of the assessment methods explicitly factors in the relational dynamic of insight assessments. For example, none include mechanisms for self-reflexivity on the side of the assessor about how confounding factors (such as social distance or the interpersonal dynamics, or other differences mentioned above) might have influenced the findings.

And yet, the terms used in relation to insight assessments, such as “agrees with,” “complies with,” “comparing to,” highlight that insight has a relational dimension which the individual-centered term “awareness of illness” obscures: insight is not simple intrapersonal “self-knowledge”; it is assessed within an interpersonal and social setting, through an interaction, which affects and co-constructs the phenomenon that is being examined.

This becomes clearer if we unpack the way insight is assessed, whether with insight scales in research or informally in clinical practice. “Assessment of insight” seems an incomplete description for what actually takes place. A better description is: establishing the nature of the awareness through communication between the individual and the clinician. Indeed, both McEvoy et al. [(16) p. 43] and David [(17) p. 799] quote Eskey’s 1958 definition of insight as “the presence of verbalized awareness…” (emphasis added). We can extend the term “*verbalized*” here to include other kinds of communication, but *because* another’s insight is something that can only be established through communication, what is, misleadingly, simply termed “awareness” must include a number of steps in order for the person to be deemed to have insight by another person:

*Step 1* A recognition—crossing the threshold of consciousness—about something in one’s experiences (*inner* *self-awareness*);

*Step 2* Making sense of the available labels and descriptions that the clinical care team presents (*cognitive capacity and socio-cultural background*);

Step 3 Connecting the labels to the experiences in oneself and understanding the implications (*self-reflexivity, and accepting a view of oneself as in grip of some undesirable characteristic*);

*Step 4* Acknowledgment of this connection in front of the clinician/care team, that is, publicly (interpersonally) accepting the connection between the experience and its medical definition (*accepting the other’s view of oneself as in grip of some undesirable characteristic*).

Each of these steps is required in order for the clinician to establish an existence of “awareness.” The steps show insight is embedded in interpersonal dynamics, dependent on trust, open communication, power balance, negotiation of language. They reveal that we currently call the patient’s “awareness of illness” is in effect *already* in some sense compliance: with a particular medical discourse presented to her as already noted above; but also with the assessment situation (the procedure, the context, the way the clinical team relate to her and describe her experience and its implications). None of the insight assessment tools has an in-built mechanism that would consider the influence of the interpersonal dynamics on how someone makes sense of their mental (ill)health.

Consider the instructions in the paper introducing the PANSS scale. It advises on the assessment procedure as follows: a semi-structured clinical interview (supplemented on some of the items, but not G12, by reports of either primary care staff or family) elicits data from the patient, which is then applied by the clinician to the 30-item form. The interview is to be, on instructions by the authors, at first “nondirective, unchallenging,” in order to establish rapport with the patient, but already in the second phase they advise the interviewer to continue with leading questions, at first “unprovocative” but then directly suggestive, such as “Do you have special or unusual powers? …Are you on a special mission from God?” The interview culminates in phase four that, the paper instructs, requires an even more “directive and forceful probing of areas where the patient appeared defensive, ambivalent, or uncooperative.” If the patient has avoided forthright acknowledgment of psychiatric disorder they are to be subjected to “greater stress and testing of limits…” and their “susceptibility to disorganization” explored [(14) p. 263]. This procedure, the authors argue, will enable the observation of, amongst other things, tension, poor rapport, uncooperativeness and hostility, cognitive disorganization, lack of flow of conversation, and anxiety, considered as some of the symptoms of schizophrenia.

It is disconcerting that PANSS procedure instructs a vulnerable person is to be subjected to “directive and forceful probing,” “greater stress and testing of limits…” and test of “susceptibility to disorganization.” First, the sought after “symptoms” such as poor rapport, tension, uncooperativeness, hostility, anxiety, or even, in extreme cases, cognitive disorganization, might be an expected response to such interpersonal stress. Second, submitting only individuals whom we perceive to have an impairment, to stringent tests about their beliefs, with the consequence of potentially coercing them into something, is problematic from human rights perspective. We all operate with certain beliefs in the background of our thinking, which not all others would share^5^. Most importantly, such approach is counterproductive in terms of assessing insight, as it affects one’s ability to think and reflect^6^. Finally, using leading questions to elicit purported evidence, in this case about lack of insight, is also problematic practice from research and legal perspectives^7^. Item G12 on PANSS is amongst the most frequently used insight assessment tools in psychiatric research (1, 41).

Is there a general problem with probing questions or could the instrument simply be deployed in a better, more sensitive way? Let us first look at an important point about the influence of a particular trait’s desirability on the tendency for a person to admit to owning this trait (24), before we return to how probing questions could be used in the vignette in the next section. Studies often try to get around the trait desirability influence by using third person vignettes, and/or scenarios where admitting a psychiatric problem brings the protagonist some kind of (financial) benefit. Despite this way around, however, in the study by David et al. (24) healthy controls were more swayed by the benefits of admitting to mental illness than participants with a diagnosis. The authors consider that a genuine inability of the people with the disorder to accurately reflect on one’s condition and/or a bias in the appraisal of material might be behind this. However, it may also be that it is easier for healthy controls to see the benefit of admitting to something that they know is not (currently) true about themselves, than for someone to whom the situation strikes too close to home. In other words, precisely the reluctance to admit might, in this case, be signaling the admission, indicating, for example, an internal conflict between the above Steps 3 and 4.

This reluctance might suggest, that the factor of dealing with stigma is affecting insight, as argued by Lysaker et al. (42). But it might also reflect something about the relationship to the assessors of insight. Even if an individual is internally aware there is something “wrong,” she might not want to own this up to the particular assessor (such as a stranger who is conducting insight research) or agree with their approach (such as a forceful probing one). Indeed, they might not be willing to “confess” that something is wrong to a medical practitioner but prefer lay persons of trust in their environment. In order that one can accept the other as someone who one is able to acknowledge things to, share, even answer to, there has to be a good relationship. Reluctance may, thus, reflect the quality of the relationship, not the level of insight. Indeed, which level of insight someone might be able to attain can depend on the nature and quality of this relationship.

Bearing in mind the above steps, particularly Step 4, someone’s insight is at least partly a product of a relational negotiation process, in which both the individual and the clinician are trying to make sense of that individual’s experience and needs. But the standard insight assessment situation projects an asymmetrical power relationship between the assessor and the assessed individual, whether in research or clinical practice context, which can skew the negotiation process. It prevents conditions that exactly underpin and enable insight, such as exploration, discovery, risk taking, testing of possibilities, time, trust. It can create resistance rather than trust and puts at the core of insight evaluation *disagreement*. Perceived disempowerment, resistance to coercion, and rejecting attempts to undermine their self-concept have been identified as some of the reasons why people reject medication [(6) p. 1464]. As Amador and Kronengold describe, disagreement can create mutual frustration, and the individual may refuse the treatment or accept it only in order to gain back their freedom. This is not specific to people with mental impairment or vulnerability; it is, the authors recognize, “what any of us would do” [(41) p. 3].

The problem of compliance is thus not yet resolved by removing treatment compliance from the insight scales; and not even by development of assessment methods that are not focused on acceptance of psychiatric language. There is an issue of compliance in the standard assessment situation itself, as summed up above in Step 4 of the assessment. This is not to suggest that it is psychiatry’s intention or aim to intimidate or control, but that it may be creating conditions with its conceptual models and procedures, where this can easily and inadvertently happen. As the authors of the study of psychiatrists’ accounts of insight put it: “We do not claim that the imposition of the medical perspective is done with the explicit goal of imposing it. Rather, it results from what could be termed the ‘professional habitus’ of the doctor, which invests the doctor with the power to impose his or her perspective” [(6) p. 1465].

In order to be able to properly understand their patients’ making sense of their experiences, a more equal relationship is required. Thus, the assessor might also have to rate themselves, their approach, the quality of their relationship to their patient, their own and patient’s social positions: variables that do not currently appear in research on insight. In other words, the assessment of someone’s self-reflexivity such as required by insight would need to include the assessor’s own self-reflexivity, in particular about how they might be influencing the very thing they are trying to measure.

The recent human rights conventions would permit the person some leeway in rejecting who will assess them and how. The CRPD and the ethical idea of autonomy for example, do grant persons—including persons with disabilities, where this includes mental illness and impairments—a say in how, where and by whom certain safeguarding procedures are implemented. Also, research context might be a slightly different case in that insight assessments are entered into voluntarily and research ethics requires that safeguards are in place. Nonetheless, as noted in the Introduction, the results from research inform classification and diagnosis of mental illness and will bear on the fate of people who did not themselves consent to be a research subject. It is arguably reasonable to require that there are further checks and balances built in. For example, Freyenhagen and O’Shea have argued that classification of mental illness should not be just left to the medical profession—because the issues at hand are not just medical but concern values about which people can reasonably disagree—but any such decisions should be scrutinized also by democratic institutions in which experts by experience should be given voice (43). Similarly, the UN’s Special Rapporteur on the right to health has called on all states to take “immediate measures to establish inclusive and meaningful participatory frameworks in the design of and decision-making around public policy” on mental health, in which service users and those in the most vulnerable situations are given a voice [(44) p. §92(a)].

We have argued in this section that it is not enough to consider that insight has been influenced by a socio-cultural context, but that it continues to be formed in myriad of social interactions and conditions, including during the insight assessment. This is something that even more inclusive notions of insight in the literature do not accurately capture. There are, broadly speaking, two main strands of insight research in psychiatry, one focusing on failures of metacognition (how a person is able to incorporate new knowledge about their abilities or deficits into their awareness of illness) (24), the other one advocating a wider approach to understanding insight (including factors such as ability to cope with stigma, social cognition and socio-cultural background) (1, 42). Both of them understand insight as something pertaining exclusively to the individual herself; they conceptualize insight “in … intrapersonal terms” [(3) p. 2].

In the next section, we analyze insight as an interactive, dynamically negotiated construct.

## Insight Constructed Relationally

A good therapeutic relationship is one of the factors consistently seen as influencing better treatment adherence, less severe symptoms, better social functioning, and fewer hospital admissions [(45) p. 517]. Psychoanalytic literature shows that positive emotional relationship with the clinician improves insight in at least some patients with psychosis (46, 47). Without a good therapeutic alliance, improved “insight” has been seen to come with the accompanying risk of deteriorating mood and quality of life of the patients, or ensuing depression (48, 49). Guidry-Grimes indicates that one reason to give more attention to insight assessments is so that more effective strategies for supporting therapeutic alliance can be suggested, this in turn leading to better goals of care [(5) p. 5].

In a sense, a good therapeutic alliance is similar to, and can perhaps be considered part of, supported decision-making. It is a relationship of trust rather than undue influence; it tries to empower rather than resort to best interest or substituted decision-making; to enable and support the person concerned to exercise their right to self-determination, to support someone to make their own decisions. This might include, for example, helping them think through their experiences, or identify their will and preferences, crucial to human rights requirements (35). This can happen through a relationship of mutual respect, where the clinician, too, can learn from the collaboration.

Aspects of the patient’s insight into particular experiences are interactively, dynamically constructed in everyday negotiations. For example, in anorexia, questions such as “Have I eaten enough?” “What is enough?” “Should I do less exercise?” “How do I feel whether it was too much exercise?” “Which situations make me compulsively walk?” “What social role does eating have for me?” etc., are discussed, interpreted with, and negotiated between the patient and the team, and other family members. Other explanations beside illness (“perhaps I am just naturally slim,” “I am strong enough to walk”) are tried, tested, sometimes defensively demonstrated, corrected. These negotiations may represent the input through which the clinician’s views of the patient’s insight develop. But importantly, these negotiations whilst affecting aspects of the patient’s insight, are also aspects of their relationship to the clinician. That means that the patient’s insight is a function, amongst other things, of this relationship. Often, patients are prepared to adhere to treatment and even to get better *for* their clinicians, and for the sake of their relationship, which they value. They eat if the clinician sits with them. They agree to face the anxiety of something, because a team member tries it with them. It might help them believe in the process if they feel the clinicians believe in them. It gives them an impetus to engage with the difficulties of the process. When at least some part of someone’s mental health difficulties might plausibly be considered to stem from their social and interpersonal circumstances, the relationship with their clinician is an opportunity for a better, more supported experience, including better ways to think about them.

It is worth considering how clinician’s self-reflexivity about their work could be included in thinking about the patient’s insight. Understanding how insight is or could be influenced by them is important. It has the potential to put the patient on a more equal footing in their relationship with the clinicians and remove the root of insight’s relation to compliance.

In the following, we summarize a vignette from Christopher Bollas’s book “When the Sun Bursts: The Enigma of Schizophrenia” (46). Bollas does not speak about insight directly, but the vignette nonetheless illustrates how insight can be supported when compliance is removed from the relationship.

Bollas describes his patient, “Lucy,” whom he worked with on the phone five times per week for several years. Lucy had an abusive childhood and bad experiences in younger adulthood. At the age of 55, she now leads a lonely, highly regulated life on a remote island in the North. She presents with hallucinations and confusion, mixing facts from her daily life with Nordic myths of her cultural background. For several years, Lucy uses her sessions to recount bad memories from her time living in a commune, but is not able to own her own narrative moments later and accuses the therapist of making things up if he tries to discuss the difficult events. Only after several years, she is able to own her recollections.

Bollas focuses his account on an episode when Lucy hallucinates a dragon that came to kill her as a punishment for something. On the phone to her therapist, Lucy runs around shouting “Go away. I did not do it. Please leave me alone” [(46) p. 192].

What is Bollas’s reaction to Lucy’s hallucination? Recalling that he has earlier mentioned to Lucy how good it was that her bad memories were not “dragging on” anymore, he puts it to her whether this might have brought an image of a dragon to her mind. At first, Lucy is furious that she is not believed the dragon is real. But the next day, she accuses him of having summoned the dragon. This interpretation is still based on a persecutory anxiety—”You said, ‘Your dragon will get you.’” [(46) p. 193]—but it signals that something from his suggestion has sank in: that dragon may have been conjured by something he had said. It is perhaps the first step to her feeling that dragon can be “controlled” by someone.

The two are then able to consider together a few thoughts in relation to the dragon: one is, that perhaps Lucy felt her therapist thought she was a drag. This made her angry, which felt to her like fire is coming out of her mouth, conjuring the image of a dragon. It helped Lucy that they were able to discuss her feelings. Another thought they consider is that the therapist was “dragging on” about things more than she could take, and thus, she was right to be upset. This acknowledgment helped Lucy, too: that the therapist was self-reflexive about his own probing questions having gone too far; that despite this probing being part of “his job,” it matters how it is done and sometimes he does not “get things right” [(46) p. 194], which also has a direct role in Lucy’s experiences. In other words, for probing questions it matters that there is in a mutual rather than asymmetrical compliance-expecting relationship: self-reflexivity from the patient needs to be matched by one from the therapist.

Bollas took Lucy’s hallucinations as expressions of her experiences that he could use to reach her: as tools for his insight into her state of mind, which then helped him support her insight into her experience. He took the content of the hallucinations and their accompanying feelings as prima-facie *valid* and looked for the “reasoning” behind experiencing certain feelings that culminated in the image of a dragon. This allowed the two of them to together gain an understanding of her inner life (insight) and for her to let go of the image.

After five years of analysis, Lucy was no longer hallucinating. Slowly, the objects in her internal world could be distinguished from the real object world, which instead “had become its own thing, not subject to anyone’s narration” [(46) p. 196]. Her experience of external reality could become close to the therapist’s one.

If we compare this to a conception of insight where the clinician insists that Lucy acknowledges that the dragon is simply not real, recognizes it as a pathological symptom, which she needs to attribute to her mental illness (schizophrenia) and take medication to get rid of it, is that the only “insight” that can be considered valid and appropriate in how she related to her experiences? Is the only “insight” one is allowed about one’s difficult experiences, a medical one?

We find that a striking and uncomfortable suggestion. Lucy was able to, with support, reflect on and process her experiences. The existing insight tools would arguably not appropriately recognize this. Approaching her with expectations that she acknowledges her experiences as symptoms and illness might even inhibit rather than support this ability in her case.

It seems hard to imagine within psychiatric discourse that the question of insight of someone who is, for example, talking about being a “victim of conspiracy to rob them of their freedom” [(2) p. 1], or, for that matter, seeing dragons trying to kill them, can be approached differently: without reference to whether they believe they are ill, or even whether their experiences track reality accurately, but with references to whether they are able to, at least with support, relate to their experiences in a self-reflexive way. Approaching the question of insight differently would also mean exploring how both parties, as well as their wider socio-cultural contexts, have had a role in the mental constructions emerging, and that this can be used to help understand the patient. More importantly still, that this can be used for the patients to understand themselves better, to gain better insight into their own experiences.

In turn, it is then hard to imagine within psychiatric discourse that someone who does not believe they are ill in compliance with the medical model, could “use and weigh” the proposed treatment appropriately to make a decision for themselves (2).

In the discourse, focused on treatment solutions, risk assessments, and planning, it is a challenging thought that all communication, including the one taking place in assessment of insight, might be multi-layered, always doing more than what is being exchanged on the surface, and always developing in the interaction. The way to health for someone like Lucy within psychiatric views on insight seems to be restricted to continuous evaluations of whether what she experiences matches the reality of others (and particularly a set of them, trained in the psychiatric model).

In their response to Guidry-Grimes’s paper raising ethical concerns around the use and conceptualizations of insight (5), David and Ariyo (3) argue that views that question insight within perspectives of ethics, philosophy, autonomy, and similar, should be distinguished from understanding insight as a phenomenon. About the latter, academics, “predisposed to question if not dispute relevant evidence and facts,” can be reassured with confidence that all the relevant systematic reviews and meta-analyses, dozens of studies on thousands of participants have been done. The relevant conclusions about clinical insight are “as close to being ‘facts’ as anything else in the clinical psychiatry” [(3) p. 1].

This view neglects the knowledge that these perspectives have brought in the last three decades *about* how people function and experience themselves. One’s experiences and one’s understanding of them, including self-knowledge, and self-reflexivity are continuously co-constructed, in any moment within real complex relationships and multiple social networks (50–52). This means that others are a fundamental dynamic part of how someone’s insight about these experiences is continually created. Of particular importance to these views is also an inclusion of temporality in understanding of a person, both in the sense that an one’s personal history has started to be understood as inherent in thinking about one’s autonomy, as well as that a person continues to be formed through relationships [(53) p. 115]. It is real relationships, histories, and socio-cultural contexts that can, when things go wrong, give rise to certain features of mental illness (54).

If a person’s experiences, including reflecting on themselves, are continuously formed through complex relational dynamics and through endless and changing discourses of power (55), then it is counterproductive to measure insight in a context permeated by requirements of compliance or as a trait of one separate individual.

It is not established that the medical conceptualization is always the most helpful one before there is data in the studies on insight about, for example, the patients’ views of their relationship with the clinician, clinicians’ reflections on where they might have got some of their treatments wrong, insight being recognized in views different from the medical one. It is thus reassuring that both, the perspectives of ethics and autonomy as well as psychiatric research understand that insight is a “biopsychosocial construct *par excellence*” [(2) p. 3]. This means that the psycho-social parts (the content of hallucinations, the socio-economic conditions correlated to particular “symptoms,” the correlation of insight to how the patient views her relationship to her clinician, etc.) need to be systematically and consistently included in insight conceptualizations and studies.

In sum, insight, particularly when understood in the broad sense as self-knowledge, has a social, relational dimension. For self-knowledge one more often than not *does* need help from others, in the sense of reference points and understanding. This help needs to be supportive, exploratory, and including self-reflectivity on both sides; and not imposing of one, medical, view. Even if often “[a]cknowledging that one is ill or impaired or in need of help … is necessary for living an authentic life” [(3) p. 2], sometimes, for living an authentic life, one first needs that someone else engages with the “reality” of their dragons and helps them to understand how and why they may have emerged, rather than focus on the divergence in their respective forms of “reality”.

Insight is an interesting and useful concept in care contexts, because it is about self-knowledge and self-reflexivity as *communicated* to others. It is an essential tool to help the clinician understand how the other person thinks, feels, and experiences things. It is essential in devising support. As Guidry-Grimes argues, it should go beyond standard biomedical views of the patient’s condition (5). We have suggested that it is a profoundly relational, dynamically negotiated understanding of one’s own experience, where the particular relationship to the clinician is a variable affecting it through their interactions. The way this happens, in all the mechanisms the mind uses such as metaphors, associations, and emotions in relation to the clinician, can be part of understanding insight.

We emphasize support as the right comportment in engaging with the efforts to gain insight. In part, this is motivated by considerations about what is best for the care of the individuals in question. But it is also motivated by concerns with human rights. The CRPD has been interpreted by its UN Committee and many others as requiring a shift from the substituted decision-making framework of best interest decisions to one of supported decision-making (56–60), whereby all people are recognized both, as needing support, even those who do not have any disabilities, *and* as agents with full legal capacity, including those with the most severe mental (or physical) illnesses and impairments. Indeed, a groundbreaking 2017 report on mental health issued by the UN’s Special Rapporteur on the right to health calls for a path of progressive realization, at the end of which there would be no more involuntary assessment and treatment (44). The broad notion of insight (as self-knowledge) would still have a role to play at the end of this development and shifting to it and to supporting it would help move us along the path towards this end goal.

## Guidelines for Insight Assessments

Insight assessment scales are mainly used in research rather than in clinical practice, but they influence the diagnostic guidelines, clinicians’ views on patients’ insight, and ensuing clinical approaches to their treatment, as well as their giving evidence in courts and tribunals.

This paper showed that expectations of compliance with treatment, clinical discourse, as well as with the procedure, context, and relationships permeate insight assessments and conceptualizations. It showed that psychiatric approaches to insight, despite a seeming variety in the field, circumscribe insight, ultimately, to medical understanding of the experiences in question. It demonstrated how this can be problematic both (a) in terms of ethics and human rights requirements and (b) counterproductive in producing self-knowledge. It has shown how someone’s insight is continuously affected by the relational dynamics, which means that the context of compliance and power asymmetry inherent in the standard insight assessment setting are particularly adversarial to supporting someone to gain insight into their experiences.

The sections above have aimed to establish that insight assessment should be with the aim and in service of supporting individuals in developing better insight into their experiences, difficulties, and needs. The insight assessment procedure should consider insight’s social and relational dimensions to identify how individuals can be thus supported, where this should include building in mechanisms of self-reflexivity on part of the assessors about these dimensions. The individual assessed should be able to choose whether they want to be assessed and by whom, and to change their assessor (ideally within a pool of qualified, accredited assessors with a plurality of views, who have been appropriately trained towards being self-reflective, supporting someone’s self-reflexivity/self-knowledge and, most importantly, doing so without undue influence), or quit the assessment process.

These requirements are meant to recognize that insight is a continuing process of developing an understanding of one’s experiences and is a profoundly relational phenomenon, embedded in personal and socio-cultural contexts. In our view, considering a person’s insight, is essentially trying to understand,

“How does this person think about and understand her experiences, and how is my relationship to them affecting this experience and understanding, and my assessment of it?”

Answering this question adequately involves a lot of knowledge about each other.

What does this mean for research on insight, since researchers presumably cannot establish such an alliance with their participants during the short time of the research study? We think it could be based on observational studies of the clinicians-patients’ relationships in practice, observing how they make sense of experiences together. Researchers could help create methods to support clinicians’ self-reflexivity and making more explicit their background thinking when assessing their patients. Studies would also need to be based on participatory research to help establish what good and bad practices are when assessing insight.

Based on these reflections, human rights-compliant guidelines for the practitioner to rely on in this exploration would need to consider the following points:

1. Scope and aim:
  - Insight assessment should be based on the broad notion of insight (insight as self-knowledge).
  - Insight assessment should be applicable to everyone, not presuppose the assessed person is mentally ill but just explore their experiences.
  - Insight assessment should be undertaken with the aim and in service of empowering and supporting the person to exercise their right to self-determination and better understand their needs and experiences.
  - The assessment should be orientated towards exploring what explanatory models the person has, how they may have been influenced by various psycho-social factors, and how they can be deployed to support the person’s understanding or ameliorate potential risks, rather than measuring compliance with the medical model of psychiatry.
  - It should allow that when someone does not agree with the specific diagnosis or treatment or the medical model of psychiatry, they might still have insight.
2. Socio-cultural background:
  - The individual’s socio-cultural background and experiences should be considered.
  - Assessors should note the socio-cultural distance between themselves and the individuals assessed, and guard against potential biases, including by being self-reflexive about this distance and its impact on the mode of engagement and conclusions drawn.
3. Therapeutic alliance:
  - The assessment should be conducted within a self-chosen therapeutic relationship that the individual considers understanding and supportive. This should include the right to change the assessor and quit the assessment process.
  - Individual’s personal circumstances, protection from social stigma, or impact on self-identity should be taken into account when assessing insight. Assessors should be mindful of the difficulty to own up publicly to a connection between one’s experiences and medical descriptions and labels.
  - Insight assessment should never constitute undue influence on the person’s responses. Assessors should be self-reflexive about how their mode of engagement might make insight-qua-self-knowledge more difficult to achieve and change up their mode of engagement accordingly.
  - Research into insight should take into account the whole relationship, rather than focusing solely on the assessed.

## Author Contributions

PC wrote the core of the manuscript, and in particular the parts about problems with compliance and the relational aspects of insight. FF contributed material on insight and ethical principles, human rights, and CRPD, and provided substantial comments on various draft versions. SG wrote about insight at the interface with the legal settings, the use of insight in Court of Protection, and the comparison of therapeutic relationships with supported decision-making.

## Conflict of Interest

The authors declare that the research was conducted in the absence of any commercial or financial relationships that could be construed as a potential conflict of interest.

## Footnotes

1 The authors of the study point out that the accounts are not in line with the part of psychiatric literature which is more insistent on working with patients’ experiences and critical of focusing only on symptoms. [(6) p. 1465].

2 The study’s authors caution that the results demonstrate psychiatrists’ discourse rather than what is done in practice.

3 By way of context, in England and Wales mental capacity—often known as mental competency in other jurisdictions like the US—is legally understood in such a way that showing that a person lacks it in relation to a specific decision at the material time the decision has to be made, requires showing (a) that the person suffers from a dysfunction or impairment of the mind or brain (i.e. meets what is often called the “diagnostic threshold”) and (b) that the person lacks one or more of four functional decision abilities, such as understanding the relevant information.

4 For example, Markova, slightly differently, proposes distinguishing between a wider notion of insight as a phenomenon and the clinical concept of insight that concerns only a small section of it (1).

5 For a good account of holding subjective beliefs that give meaning and enable our “cultural” existence, and exist in the paradox of simultaneously not to be questioned but also not to be imposed on the others, see (36).

6 Not only in a vulnerable person but in most people. Clare Lamontagne gives an example of how an intimidating supervisor adversely affects nurses’ thinking and creates confusion in administering medication. [(37) p. 59]. Reyes et al. (38) show that stress in the form of interpersonal challenge decreases metacognitive sensitivity.

7 See, for example, Bowles and Sharman (39) and Perlman et al. (40) on the effects of using leading interview questions in court with vulnerable adults.
